# Identification and validation of an m7G-related lncRNAs signature for predicting prognosis, immune response and therapy landscapes in ovarian cancer

**DOI:** 10.3389/fgene.2024.1466422

**Published:** 2024-10-08

**Authors:** Jixin Li, Hui Wang, Siyang Zhang, Linru Quan, Xin Zhou

**Affiliations:** Department of Obstetrics and Gynecology, Shengjing Hospital of China Medical University, Shenyang, Liaoning, China

**Keywords:** ovarian cancer, N7-methylguanosine (m7G), long non-coding RNA (IncRNA), prognostic signature, immune response

## Abstract

**Background:**

Ovarian cancer is the most mortality malignancy in gynecology. N7-methylguanosine (m7G) is one of the most prevalent RNA modifications in the development and progression of cancer. The aim of this study is to investigate the effect of m7G-related lncRNA on ovarian cancer in terms of instruction prognosis and immunotherapy.

**Methods:**

After integrating and processing the RNA expression profiles with the clinical sample information in the TCGA database, we initially screened to the m7G-related lncRNAs by Spearman correlation analysis, and subsequently obtained a prognostic model constructed by five m7G-related lncRNAs with Univariate Cox analysis, LASSO regression analysis, and Multivariate Cox regression analysis, after which we further evaluated and validated the prognostic value of the model using Kaplan-Meier survival analysis, Principal component analysis, Nomogram, and ROC curve. In addition, based on this risk model, we explored the differentially enriched pathways and functions of the high and low risk groups, and characterized the immune cells, immune functions, gene mutations, and drug sensitivity between the two groups.

**Results:**

After a series of rigorous filtering, we finally attained a prognostic risk model consisting of KRT7-AS, USP30-AS1, ZFHX4-AS1, ACAP2-IT1, and TWSG1-DT which is excellent in predicting the prognostic survival of ovarian cancer patients as well as existing as an independent prognostic factor. Moreover, the model has certain relevance in the immune cells and functions between high and low risk groups, and simultaneously, the signature has the role of guiding the option of immunotherapy and chemotherapeutic drugs.

**Conclusion:**

Altogether, our study established a tight connection between m7G-associated lncRNAs and ovarian cancer, with potential that the prognostic patterns contribute to steering the prognosis of ovarian cancer patients, measuring the efficacy of immunotherapeutic approaches, and detecting effective chemotherapeutic agents.

## 1 Introduction

Ovarian cancer (OC) is among the gynecologic malignancies with the highest mortality rate and the worst prognosis for heterogeneous disease (Xiao et al., 2022). Annually, 2,00,000 women worldwide are diagnosed with ovarian cancer, and the majority of patients are diagnosed at an advanced stage (Foster et al., 2023). Being the fundamental of contemporary ovarian cancer management, the standard of care for ovarian cancer consists of initial tumor cytoreductive surgery followed by platinum-based chemotherapy, or intermediate tumor cell reduction surgery performed after neoadjuvant chemotherapy, and postoperative chemotherapy (Xiao et al., 2022; Foster et al., 2023). However, the majority of patients experience postoperative recurrence and chemotherapy resistance, which results in a 5-year survival rate of less than 40% for ovarian cancer, requiring considerable effort on the part of researchers, and there is an urgent need to develop novel therapeutic strategies, with in-depth molecular analyses of OC to assist in guiding more precise and personalized clinical decision-making (Xiao et al., 2022; Hosseininasab-Nodoushan et al., 2022; Konstantinopoulos and Matulonis, 2023).

Unlike genetic alterations, which change the DNA sequence, epigenetics works by manipulating the gene expression processes that promote tumorigenesis and which aid in the acquisition of oncogenic capacity (Davalos and Esteller, 2023). RNA modification is an imperative component of epigenetics and m7G, the most common type of RNA modification, refers to N7 position guanine RNA methylation, which was first identified at the 5’cap of mRNAs, to either stabilize and further mobilize transcripts or interfere with the biological functions associated with caps (Liu et al., 2024; Ramanathan et al., 2016). The m7G can affect almost the entire process of RNA metabolism, including stabilization of mRNA structure, shearing, transcription, translation and nuclear export (Luo et al., 2022). The most common site for m7G modification is at nucleotide position 46 in the variable loop region of tRNA (Zhang et al., 2019). Moreover, m7G modifications can also be observed in the internal sites of mRNAs, tRNAs, and rRNAs, where the modifications exert roles in regulatory shearing of pre-mRNA, nuclear translocation, and translation; stabilize the structural intactness of tRNAs to accelerate translation and minimize ribosomal pausing; and are involved in the biogenesis of the 18 S rRNA precursor and in the nuclear export of 40 S rRNAs (Luo et al., 2022; Zhang et al., 2019; Haag et al., 2015; Lin et al., 2018; Galloway et al., 2021).

lncRNA, a non-coding RNA more than 200 nucleotides in length, has attracted the attention of a wide range of researchers in recent years, and although it does not encode proteins, it nonetheless can serve vital functions in the progress, metastasis and prognosis of cancer (Olgun et al., 2018; Cheng and Steinrücken, 2023). As well as governing gene transcription by acting as recruiters and scaffolds with regulatory factors participating in epigenetic modifications, lncRNAs can also behave as co-regulators or repressors affecting gene expression. Furthermore, lncRNAs can function as sponges for miRNAs, which prevent their action and in turn restrict processes like mRNA machining, stabilization, and translation (Chen et al., 2022; Chillón and Marcia, 2020; Kung et al., 2013). There are available data suggesting that a novel negative malignant pleural mesothelioma prognostic marker, Linc00941, and exposing the underlying features of this lncRNA in the attainment of aggressive characteristics by malignant pleural mesothelioma cells (Gugnoni et al., 2024). In the colon cancer, the differentially highly expressed lncRNA RP11-197K6.1, which was negatively correlated with patient survival, facilitated the migration and metastasis by interacting with miR-135a-5p to foster tumor growth (Wang et al., 2024). The m7G-modified LncRNA TEKT4P2 and LncRNA DNM1P41 can have a pivotal influence in the HBV-mediated inflammatory-oncogenic transformation of Hepatocellular Carcinoma (Shi et al., 2023).

In view of the latest research progress in LncRNA and m7G modification, it is reasonable to assume that the construction of m7G-related lncRNA prognostic model may be instrumental in guiding the prognosis of ovarian cancer patients, screening for effective small molecule therapeutic agents, and evaluating the efficacy of immunotherapy. Hence, this study will focus on the above aspects to address the prognosis, immune response and therapeutic outlook of ovarian cancer.

## 2 Material and methods

### 2.1 Data acquisition and manipulation

The RNA-seq profiles for ovarian cancer samples from the TCGA database and normal ovarian samples from the GTEx database were downloaded from the UCSC Xena database (https://xena.ucsc.edu/), and corresponding mutation data and clinical information were extracted from the corresponding databases (accessed 22 May 2023). After checking the clinical data to exclude patients who lacked information on survival time and status, the final cohort included in the study consisted of 374 ovarian cancer samples and 88 normal ovarian cases. The transcriptome and clinical data of the ovarian cancer OV-AU dataset were retrieved from the International Cancer Genome Consortium (ICGC) database (https://dcc.icgc.org/) (accessed on 16 October 2023) as an external validation dataset for subsequent prognostic modeling, and samples lacking in survival time and status were removed, and a total of 81 samples of ovarian cancer from the ICGC cohort were ultimately enrolled in the study. The annotation of transcriptomic data was performed using the Human Genome Annotation file (GRCh38.110) from the Ensembl website (https://asia.ensembl.org/index.html), and mRNA and lncRNA expression matrixes were isolated.

### 2.2 Identification of m7G-related lncRNAs

A list of 40 m7G regulators available according to the Molecular Signatures Database (MSigDB) Team (https://www.gsea-msigdb.org/gsea/msigdb) and the published literatures is summarized in Supplementary Table S1. Differential expression analysis of lncRNA profiles of ovarian cancer samples and normal ones was performed using the “DESeq2” package in the R program with screening criteria: |log2FC|>1, False Discovery Rate (FDR) < 0.05. Then, differentially expressed lncRNAs were collected, and the results of variance were visualized by volcano plots using the “ggplot2” package. Subsequently, the spearman correlation analysis was applied to calculate the correlation coefficient between m7G-related genes and differentially expressed lncRNAs to identify m7G-related lncRNAs, with the following criteria: |correlation coefficient| >0.4, P< 0.05.

### 2.3 Construction prognostic signature of m7G-related lncRNAs

Integrating the survival data, a univariate Cox analysis of m7G-related lncRNAs was carried out using the “survival” R package, and the Hazard Ratio (HR) was calculated for each lncRNA, with those that met the criteria being analyzed in the next stage (*P* < 0.05). To reduce overfitting, LASSO regression analysis was utilized for the prognosis-related lncRNAs obtained in the prior stage using the “glmnet” R package, and the lambda. min was considered as the threshold to further shrink the lncRNAs. Then we performed Multivariate Cox regression analysis on the acquired lncRNAs and constructed a prognostic signature for ovarian cancer patients based on m7G-related lncRNAs with the model formula:

$$Risk\;Score=\sum_{i=1}^{n}Expri\times Coefi$$

(where n denotes the number of modeled lncRNAs, Expri and coef_i_ represent the expression of each lncRNA and its corresponding coefficient, respectively.) Thereafter, the Sankey diagram was graphed to reveal the degree of correlation between m7G prognosis-associated lncRNAs and their corresponding mRNAs.

### 2.4 Evaluation and validation of the prognostic model of m7G-related lncRNAs

Based on the above equation, risk score was calculated for each patient in the TCGA cohort and in the external dataset employed to validate the generalizability of m7G-related lncRNAs prognostic model, and patients were categorized into high- and low-risk groups in terms of median risk score. The clinicopathological characteristics of patients with OC are extracted of training and validation groups in Tables 1, 2. Afterwards, Kaplan-Meier survival analysis was undertaken to compare the differences in overall survival (OS) between the two groups on the basis of the “survival” and “survminer” R packages. The “timeROC” R package was used to plot the Receiver Operating Characteristic (ROC) curve, and the Area Under the Curve (AUC) was calculated to determine the accuracy of the model in predicting the survival of ovarian cancer patients. In the following, we validated the prognostic model in the ICGC database by adopting the above methods for further verifying the accuracy of the signature.

**TABLE 1:** Clinicopathological characteristics of OC patients in TCGA.

Characteristics	Category	n (%)
Age	≤60	205 (54.8)
	>60	169 (45.2)
Status	Alive	145 (38.8)
	Dead	229 (61.2)
Stage	I	1 (0.3)
	II	22 (5.9)
	III	291 (77.8)
	IV	57 (15.2)
	unknow	3 (0.8)
Grade	G1	1 (0.3)
	G2	42 (11.2)
	G3	320 (85.6)
	G4	1 (0.3)
	unknow	10 (2.7)

**TABLE 2:** Clinicopathological characteristics of OC patients in ICGC.

Characteristics	Category	n (%)
Age	≤60	47 (58.0)
	>60	34 (42.0)
Status	Alive	15 (18.5)
	Dead	66 (81.5)
Stage	III	69 (85.2)
	IV	12 (14.8)

To efficiently assess the ability of m7G-related lncRNAs prognostic model to distinguish between OC patients with various risk scores, we employed principal component analysis (PCA) for validation with the assistance of the limma and scatterplot3d packages. In addition, univariate and multivariate Cox regression analyses were chosen to appraise independent prognostic factors of OS, including risk score, age, grade, and stage. Finally, the correlation between risk score and clinicopathologic characteristics was demonstrated accordingly.

### 2.5 Construction of predictive nomogram

In order to fully utilize this prognostic model, a nomogram was constructed by combining risk scores, age and clinical stage making use of the “rms” R package. In the prognostic nomogram scoring system, each variable was assigned a corresponding score, and the sum of the scores for all variables for each patient was the total score, which was calculated to predict the patient’s probability of survival at 1, 3, and 5 years. Moreover, calibration curves for the 1-, 3-, and 5-year were plotted to assess the accuracy of the prognostic nomogram. If the calibration curve is nearly 45°, the model built with the above factors has considerable predictive capability. Likewise, Decision curve analysis (DCA) with dependence on the R package (dcurves and ggplot2) was analyzed for assessing the net benefit of OC patients at different threshold probabilities, which in turn led to an attempt to gauge the prophetic capacity of the risk model.

### 2.6 Functional enrichment analysis

Differentially expressed genes (DEGs) between low and high risk groups were probed using the “limma” software package with thresholds of FDR <0.05 and |log2FC| ≥ 1. After that, Gene Ontology (GO) and Kyoto Encyclopedia of Genomes (KEGG) were enriched and analyzed for potential differentially abundant biological functions including molecular function (MF), biological process (BP), cellular component (CC) and related pathways between the two risk groups employing the R software package “clusterProfiler” and “edgeR.”

### 2.7 Calculation of the tumor mutation burden and somatic mutation analysis

After obtaining the variation data of OC patients, the somatic mutation was analyzed utilizing a “maftools” package according to the established risk signature between groups. An association of tumor mutational burden (TMB) relating to genomic instability and immunogenicity involving base substitutions, gene insertions and deletions, and other mutations holds the promise of being predictive of prognosis and the effectiveness of immunotherapies (Huang et al., 2021). In conjunction with the risk scores, TMB was estimated by corresponding R package and visualized these results in degrees.

### 2.8 Immune cell and immune function analysis

To exploit the predictive precision of the prognostic model of m7G-associated lncRNA for immunotherapy in OC patients, three algorithms, CIBERSORT, ESTIMATE and ssGSEA, were chosen to investigate the relationship between the signature and immunity in this study. CIBERSORT is an instrument for analysis of expression data to indicate the composition of cells in complex tissues based on pre-processed gene expression profiles, and to assess the relationship between risk scores and immune cells using Pearson correlations (Newman et al., 2015). The CIBERSORT algorithm was performed on the high and low risk groups of the TCGA cohort to calculate the relative percentages of 22 immune cells in ovarian cancer samples. The immune score, stromal score, estimate score, and tumor purity were determined using the ESTIMATE program and the relevant boxplots and the bar chart were drawn to present the results more clearly (Yoshihara et al., 2013). The ssGSEA method was used to determine the degree of infiltration of immune functions and further compare the difference between the two groups in terms of the expression of immune checkpoints, and the results were visualized using the “ggplot2” R package (Barbie et al., 2009).

### 2.9 Immunotherapy analysis and drug sensitivity prediction

The tumor immune dysfunction and exclusion (TIDE) (http://tide.dfci.harvard.edu/) algorithm can be implemented to access the effectiveness of immunotherapy by comparing the expression of immune checkpoint inhibitors (ICIs) between the two groups and further forecasting the OC patients’ response to immunotherapy by utilizing the corresponding R-packages. The half-maximal inhibitory concentration (IC50)is the concentration that can cause 50% of a certain effect in a subject, and it is a major indicator for evaluating the efficacy of an agent or the response of a sample to a treatment. We obtained the relevant data from the database, and utilized the “pRRophetic” R package to calculate the IC50 of the anticancer agents in patients of the TCGA cohort, and to estimate the curative effects of the different chemotherapeutic drugs.

### 2.10 Development of the ceRNA network associated with the prognostic signature of m7G-related lncRNAs

We constructed ceRNA regulatory networks that necessitate m7G-associated prognostic models, m7G regulators, and miRNAs that interact with them. The miRNAs interacting with the prognostic signature of m7G-associated lncRNAs and those reacting with m7G regulators were retrieved from the Lncbase online site (https://diana.e-ce.uth.gr/lncbasev3) and miRWalk website (http://mirwalk.umm.uni-heidelberg.de/), respectively. Crossing the prediction miRNAs resulted in the formation of lncRNAs-miRNAs-mRNAs ceRNA network, which was visualized by cytoscape software.

### 2.11 RNA isolation and quantitative reverse transcriptase PCR (qRT-PCR)

Total tissue RNA was extracted from 12 OC samples including six in early phase and six in late phase using Trizol reagent in accordance with the manufacturer’s instructions. The cDNA synthesis was performed here starting with the PrimeScript™RT reagent Kit with gDNA Eraser (Code No. RR047A; Takara). The qRT-PCR analysis was completed utilizing the TB Green premix Ex Taq^TM^ II(Code No. RR820A; Takara), following the procedure in the description. The relative gene expression was calculated by the -ΔCt method, in which Actin was taken as an internal reference. The primer sequences applied are summarized in Supplementary Table S2.

### 2.12 Statistical analysis

This study was primarily analyzed statistically using R software (version 4.3.1) and the associated R packages, and *p*-values <0.05 were considered statistically different. The prognostic value was evaluated by Cox regression. The glmnet package was used to calculate the optimal penalty parameter lambda and the related coefficient criterion of the Lasso Cox regression algorithm. Spearman correlation analysis was used to analyse the correlation between m7G-related genes and differentially expressed lncRNAs. The overall survival of KM survival curves was calculated by the log-rank test. The predictive accuracy of the prognostic model for OS was evaluated by performing ROC curve analysis. Differences in the proportions of clinical characteristics were analysed by the chi-squared test. The mRNA levels of m7G-related lncRNAs were determined using the Mann–Whitney U test. The degree of difference was noted: * if *p* < 0.05, ** if *p* < 0.01, *** if *p* < 0.001 and **** if *p* < 0.0001.

## 3 Results

### 3.1 Filtering of m7G-associated lncRNAs in ovarian cancer

**FIGURE 1:**
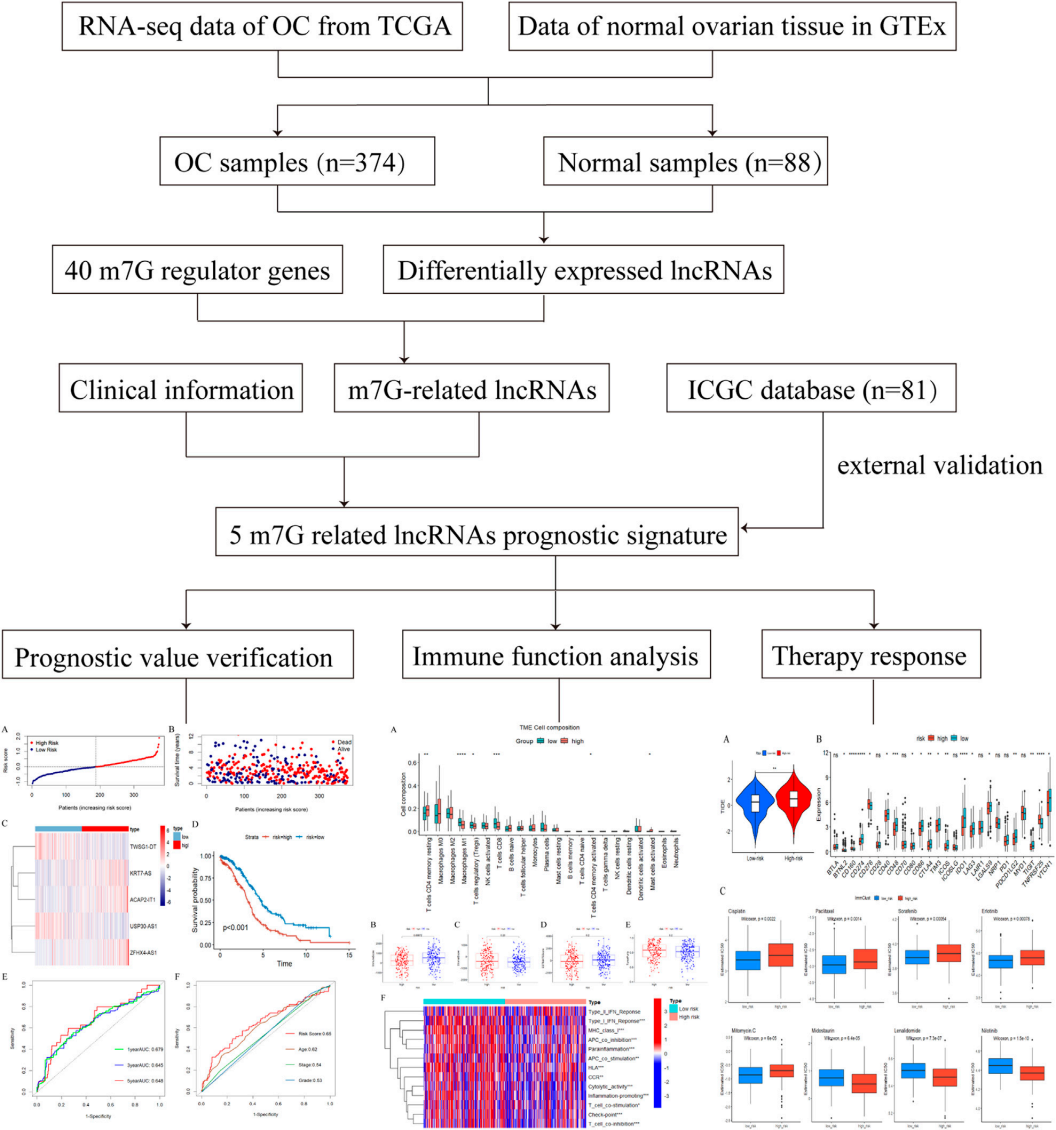
The flowchart of the detailed study.

**FIGURE 2:**
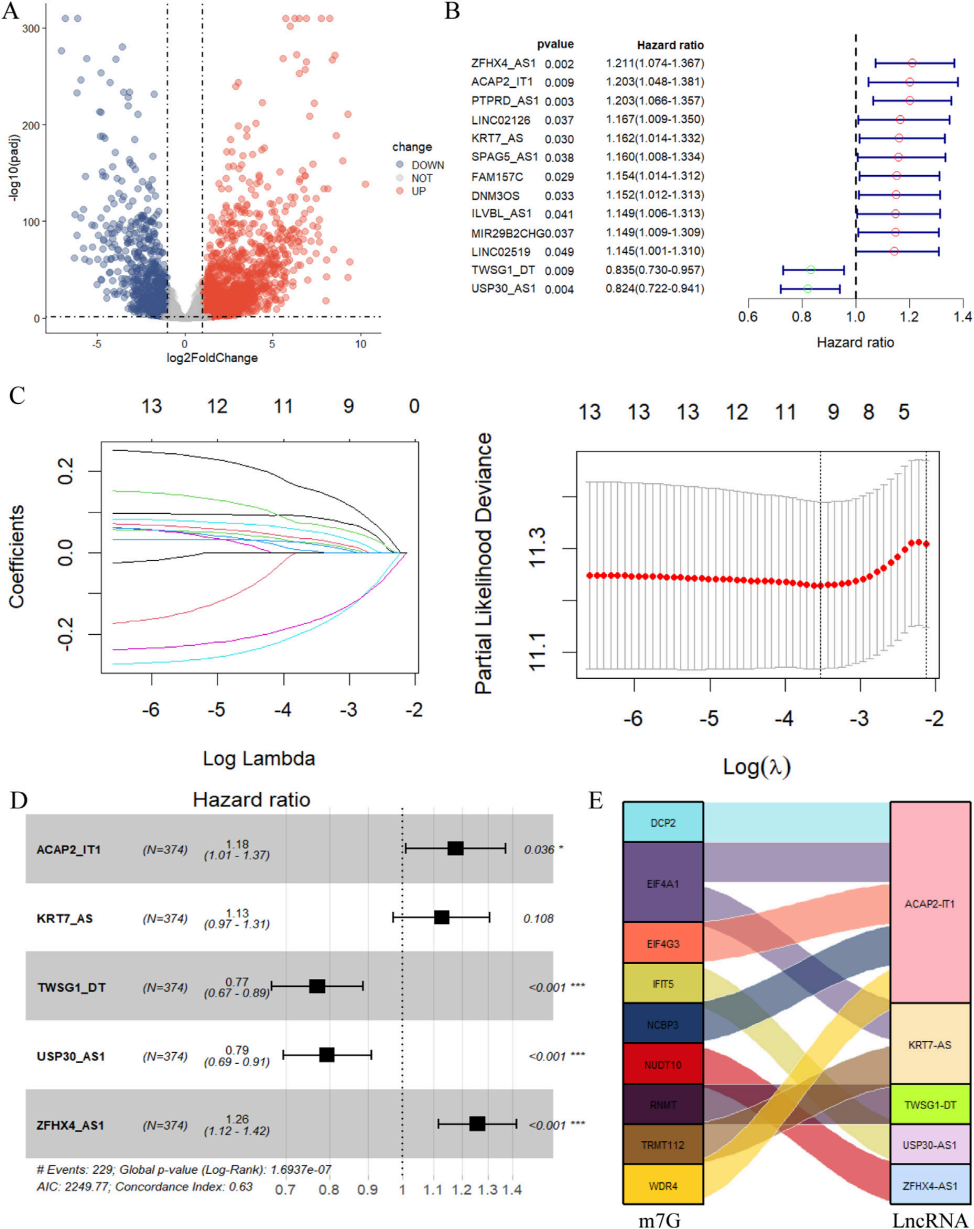
Construction of the m7G-related lncRNA prognostic model. **(A)** The volcano plot of differential expression lncRNA. (|log2FC|>1, FDR <0.05. Red dots represent differentially highly expressed genes, and blue dots represent differentially low expressed genes.) **(B)** The 13 prognostic m7G-related lncRNA derived from univariate Cox regression analysis (P< 0.05). **(C)** Cvfit and lambda curves showing the least absolute shrinkage and selection operator (LASSO) regression was performed with the minimum criteria. **(D)** The 5 prognostic m7G-related lncRNA selected by multivariate regression analysis (*p < 0.05, **p < 0.01, ***p < 0.001). **(E)** The relationship of 9 m7G-related genes and 5 lncRNA visualized in Sankey diagram (|correlation coefficient| >0.4, *P* < 0.05).

A flowchart of the entire research is presented in Figure 1. After data processing, we analyzed the differentially expressed lncRNAs in the TCGA database by means of the “DESeq2” package, and screened out a total of 2079 differentially expressed lncRNAs, of which 1,403 were upregulated and 667 were downregulated. The differentially expressed lncRNAs were summarized in a volcano plot prepared with the “ggplot” package (Figure 2A). The Spearman correlation analysis was subsequently performed on the 40 m7G regulators and the differentially expressed lnRNAs mentioned above, with the screening criteria as described herein, and a total of 257 m7G-associated lncRNAs were ultimately identified in ovarian cancer (Supplementary Figure S1).

### 3.2 Construction of an m7G-related lncRNA-based prognostic model

The 257 m7G-related lncRNA expression matrix of ovarian cancer was merged with the clinical information, and 13 m7G-related lncRNAs that were significantly associated with the prognosis of ovarian cancer patients were firstly sieved by the univariate Cox regression analysis (Figure 2B). In the next step, these 13 prognosis-related lncRNAs were subjected to LASSO regression analysis to eliminate certain lncRNAs that might lead to overfitting, and lambda. min was selected as the threshold, and a total of 10 m7G-related lncRNAs were identified by the screening (Figure 2C). This was followed by multivariate Cox regression analysis, and finally 5 m7G-related lncRNAs associated with ovarian cancer prognosis were shortlisted for the establishment of the prognostic prediction model (Figure 2D). These five m7G-related prognostic lncRNAs were KRT7-AS, USP30-AS1, ZFHX4-AS1, ACAP2-IT1, and TWSG1-DT, among which KRT7-AS, ZFHX4-AS1, and ACAP2-IT1 were risk factors for ovarian cancer (HR > 1), while USP30-AS1, and TWSG1-DT were protective factors (HR < 1), and Sankey diagrams more intuitively demonstrated the above results (Figure 2E). The equation for this prognostic model is:

Risk Score = 0.121·expr (KRT7-AS) - 0.231·expr (USP30-AS1) + 0.231·expr (ZFHX4-AS1) + 0.163·expr (ACAP2-IT1) - 0.261·expr (TWSG1-DT).

### 3.3 Validation of the prognosis signature of m7G-related lncRNA

**FIGURE 3:**
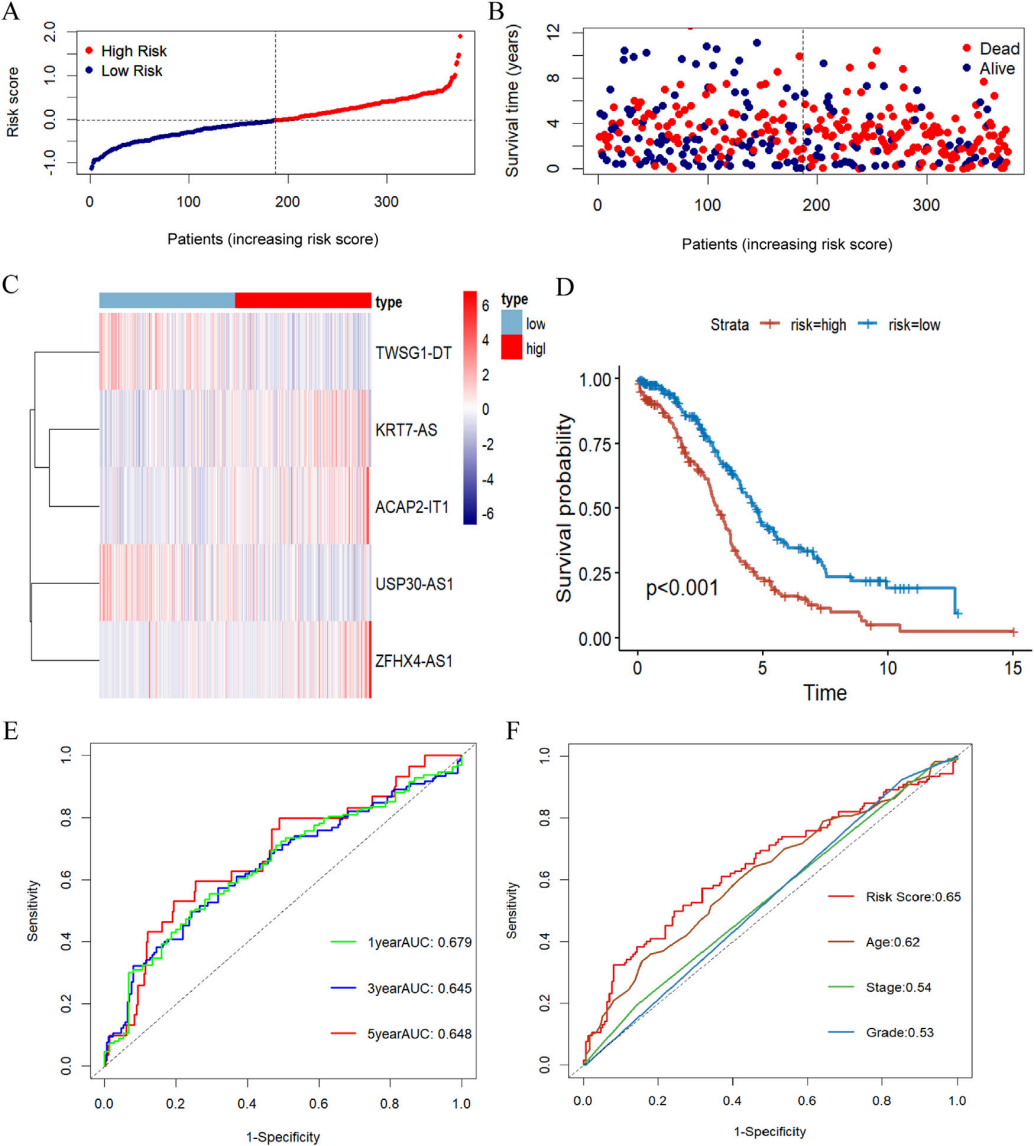
Evaluation of the prognosis signature of m7G-related lncRNA. **(A)** Risk score distribution of OC patients in the TCGA database. **(B)** The survival status for OC samples in the test group. **(C)** The heatmap displaying the differential expression of the five prognostic m7G-related lncRNAs in the high- or low-risk group. **(D)** Kaplan-Meier survival curve with log-rank test demonstrating survival analysis of five m7G-associated lncRNA prognostic signature between high- and low-risk groups in the training set. **(E)** The ROC curve showing the potential of the prognostic m7G-related lncRNAs signature in predicting 1-, 3-, and 5-year overall survival in the training group. **(F)** AUC of ROC curves evaluating the prognostic precision of risk score and other clinicopathologic features in the test group.

Aiming to evaluate the prognostic effect of the signature, the above risk score formula was implemented in different datasets. Then, we selected the TCGA database as the training set and the ICGC database as the validation set, and calculated the risk score for each sample according to the above formula, and categorized the patients into high and low risk groups based on the median risk score. As shown in Figures 3A–C, which more closely visualizes the distribution of characteristic risk scores, survival status, and associated lncRNA expression in the training set. With higher risk scores, more deaths occurred in patients, which indicates that the prognosis of patients at higher risk levels is inferior. In addition, a significant difference in the expression levels of the lncRNAs constituting this prognostic model was also observed between the two groups of patients, with KRT7S-AS, ZFHX4-AS1, and ACAP2-IT1 being expressed more in the high-risk group than in the low-risk group, whereas USP30-AS1 and TWSG1-DT were higher in the context in the latter group than in the high-risk group.

Kaplan-Meier survival analysis disclosed a striking variation in overall survival between the high- and low-risk groups, with patients in the high-risk group having considerably lower overall survival than those in the low-risk group (Figure 3D). To assess its plausibility, ROC curves were organized to analyze the model with AUCs of 0.679, 0.645, and 0.648 at 1, 3, and 5 years, respectively (Figure 3E). These results indicated that the prognostic model constructed in this study for m7G-associated lnRNA in ovarian cancer had excellent predictive power. After that, we picked 3-year overall survival with less elevated AUC for the next analysis, and the risk score based on 5 m7G-associated-lncRNAs had a substantially more favorable AUC for predicting 3-year overall survival than the other clinical parameters, demonstrating that the prognostic prediction model was fairly dependable (Figure 3F).

**FIGURE 4:**
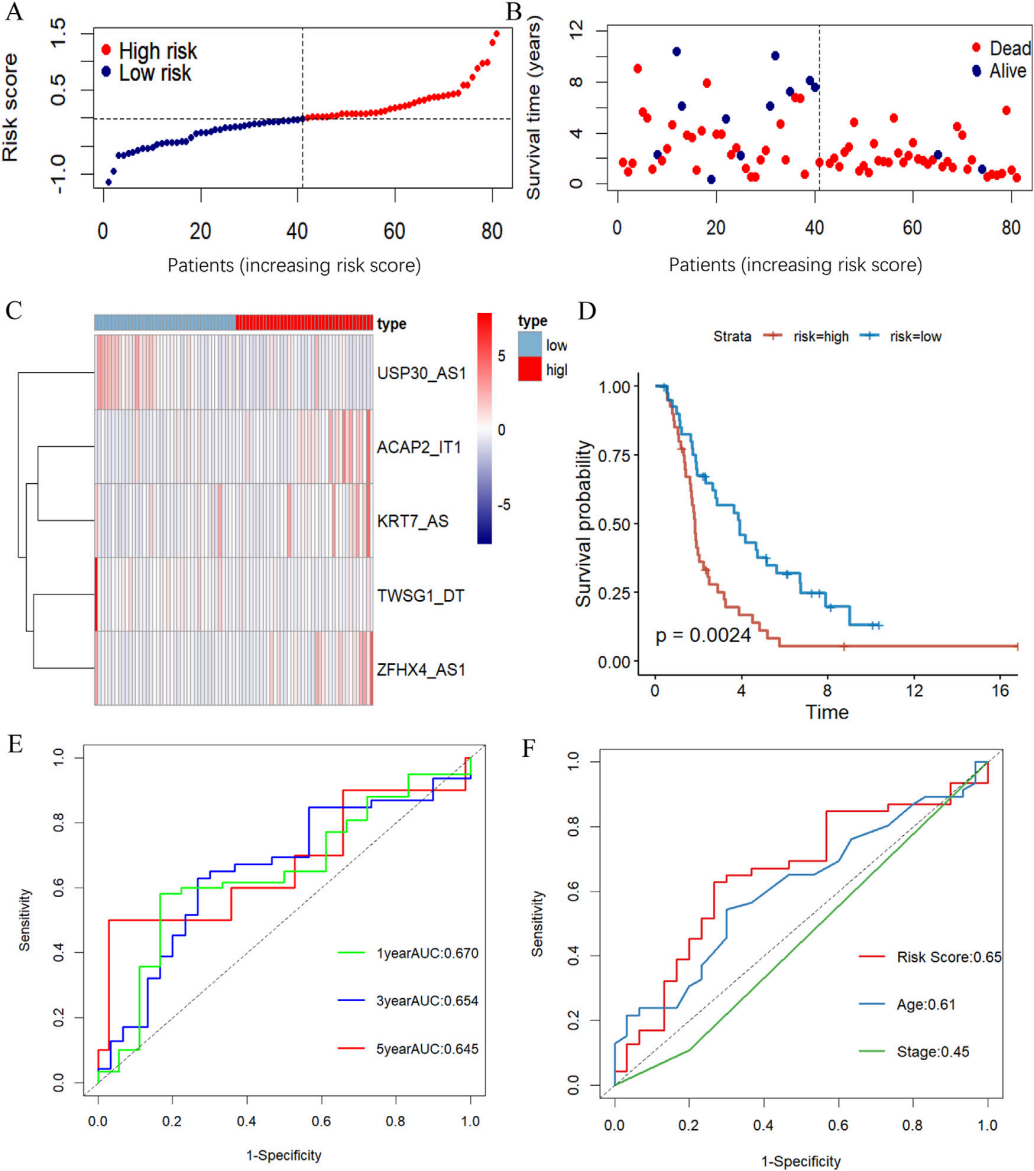
Validation the m7G-related lncRNA prognosis model. **(A, B)** The risk score distribution **(A)** and survival status **(B)** of OC patients in the validation dataset. **(C)** The heatmap illustrating the expression of five m7G-associated lncRNAs differently in the high- and low-risk groups. **(D)** KM survival curve showing survival analysis of five m7G-related lncRNA prognostic signatures between high- and low-risk groups in the ICGC database by log-rank test. **(E, F)** ROC curve illustrating the value of the prognostic m7G-associated lncRNAs signature in estimating 1-, 3-, and 5-year OS **(E)** and demonstrating the prognostic accuracy of risk scores and other clinicopathologic traits in the validation group **(F)**.

To verify the generalizability of the prognostic model, we conducted parallel experiments in an external dataset, the ICGC database, and obtained similar experimental outcomes. The distribution of risk scores and survival states and the differentially expressed situation of the model in the validation dataset were as presented in Figures Figure4A–C. In the validation group, the Kaplan-Meyer survival analysis similarly agreed that the overall survival of patients in the high-risk group was substantially lower than that of the low-risk group (Figure 4D). The effectiveness of the model in the validation group was also supported by the ROC curve analysis with AUC of 0.670, 0.654 and 0.645 at 1, 3 and 5 years, respectively (Figure 4E). And, the prognostic model also remains with excellent prognostic accuracy in comparison to other clinicopathologic features of AUC(Figure 4F).

### 3.4 Identification of independent prognostic factors

**FIGURE 5:**
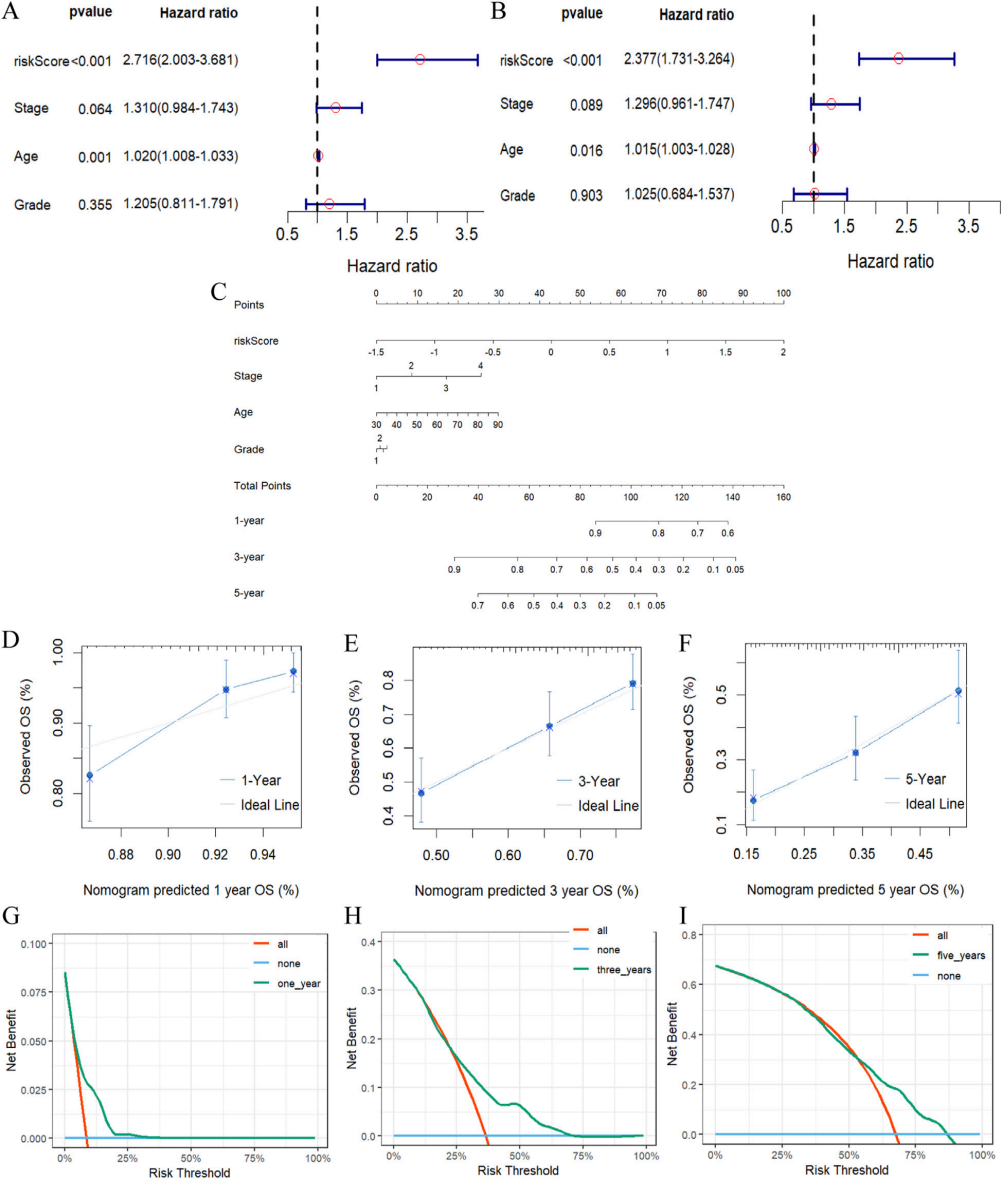
Identification of independent prognostic factors. **(A, B)** Forest plots of Univariate **(A)** and multivariate **(B)** Cox regression analysis for assess the prognostic effect of risk score and other clinical characteristics. **(C)** Nomogram for predicting 1-, 3-, and 5-year overall survival rates in patients with ovarian cancer based on m7G-associated lncRNA risk score and other clinicopathologic features. **(D–F)** Calibration curves indicating the consistency of predicted and observed rates of 1-**(D)**, 3-**(E)**, and 5-**(F)** year overall survival for patients with ovarian cancer based on nomograms. **(G–I)** The DCA curve of nomogram estimating the accuracy of the 1-year **(G)**, 3-year **(H)**, 5-year **(I)** survival rate for m7G-related lncRNAs signature.

An evaluation of whether or not the computed scores from the risk model on the basis of five m7G-associated lncRNAs could be regarded as an independent prognostic index for patients with ovarian cancer was accomplished using univariate Cox regression analysis and multivariate Cox regression analysis. Univariate Cox regression analysis results prompted that both age (HR: 1.02%, 95% CI: 1.008–1.033, *p* = 0.001) and m7G-related lncRNA risk score (HR: 2.716%, 95% CI: 2.003–3.681, *p* < 0.001) were dramatically associated with overall survival (Figure 5A). At the same time, multivariate Cox regression analysis also disclosed that age and m7G-associated lncRNA risk score were independent prognostic factors for OC patients, confirming that other clinical parameters had no consequence for the model (Figure 5B).

### 3.5 Establishment of predictive nomogram

Risk score, clinical stage, grade and age were integrated to construct a nomogram to predict the probability of survival at 1, 3, and 5 years for patients with ovarian cancer (Figure 5C). In the predictive nomogram, the lower the patient’s total score the better the patient’s prognosis, for clinical application, we can preliminarily estimate the patient’s 1-year, 3-year and 5-year survival states depending on the patient’s overall score. Besides, the calibration curve was close to the reference line, which indicated that the nomogram was of better predictability for the survival time of patients with ovarian cancer (Figures 5D–F). Combined with the probability of agreement between predicted and actual outcomes, the decision curve analysis served to estimate the independent factors’ predictive ability, and the results illustrated that the accuracy of this feature was superior to all other conventional clinicopathologic characteristics (Figures 5G–I). Collectively, the 5-m7G-related-lncRNA risk model possessed remarkable prognostic benefits in OC patients.

### 3.6 Clinical characteristics and TMB in relation to prognosis of OC

**FIGURE 6:**
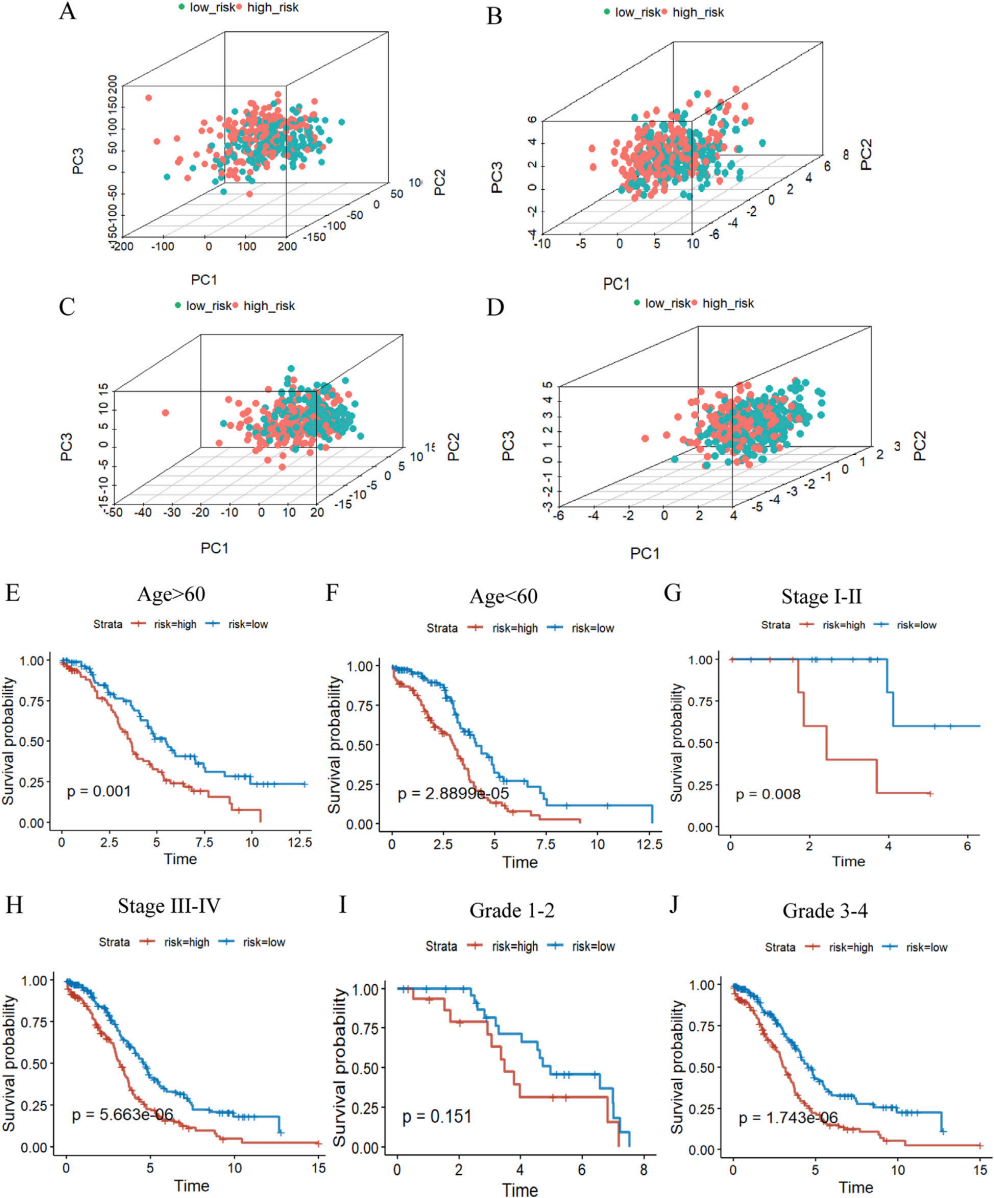
The value of the m7G risk signature. **(A–D)**, PCA based on the high- and low-risk groups to perform the distribution of total gene **(A)**, m7G regulators **(B)**, m7G-related lncRNAs **(C),** and 5 m7G-related risk lncRNAs **(D)**. **(E–J)** KM curves of overall survival stratified by age**(E, F)**, stage**(G, H)** and grade**(I, J)** between high-/low-risk groups in the whole groups by log-rank test.

The impact of this prognostic model on the difference in the distribution of the high and low risk groups was further judged by PCA in the TCGA database, as indicated in Figures 6A–D, the signature can well differentiate the sample distribution between two groups. Afterwards, we considered the association between risk scores and clinicopathological characteristics as well as prognosis toward the evaluation of the consequences of our prognostic risk model for individuals with OC, which we stratified in accordance with the clinicopathological characteristics of the patients. The findings substantiated that there were significant differences in various clinical information among the different groups except for grade1-2, which may be attributed to the fact that ovarian cancer is detected at a later stage, and the number of patients with grade 1–2 is small and unrepresentative, and the prognosis of OC patients could still be favorably differentiated based on risk scores (Figures 6E–J).

Moreover, we analyzed the mutation rates of genes in the high- and low-risk groups to further inquire into the implication of genetic mutations (Supplementary Figures S2A, B). Our results have shown that there is a difference in TMB between the high and low risk groups and that TMB is lower in the high risk group in comparison (Supplementary Figure S2C). We then analyzed the differences in overall survival between the groups using the TMB score to classify the samples into high TMB(H-TMB) and low TMB(L-TMB) groups, and the low TMB group presented a substantially reduced OS and worse prognosis compared to the high TMB group (Supplementary Figure S2D). Subsequently, in conjunction with the high and low risk groups described earlier, within the analysis of the four groups, there was also a significant variation in OS, where L-TMB and high risk signaled a terrible prognosis which points to the predominant role of risk scores in determining the prognosis (Supplementary Figure S2E).

### 3.7 Evaluation of immunotherapy benefit

**FIGURE 7:**
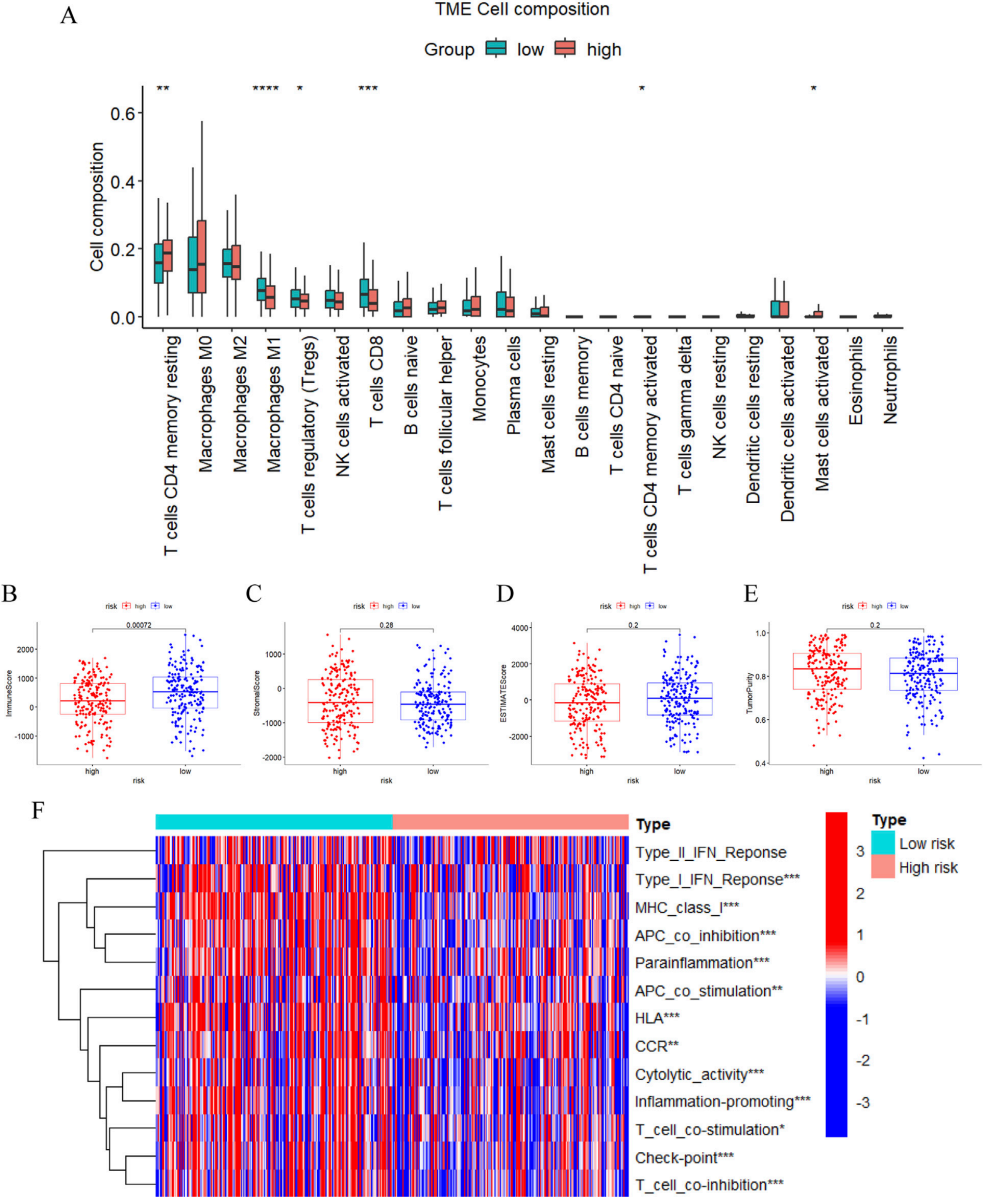
Immune cells and functions analysis. **(A)** The relative proportions of 22 infiltrating immune cells between two risk subgroups by CIBERSORT. **(B–E)** The difference of the immune score **(B)**, stromal score **(C)**, estimate score **(D)**, and tumor purity **(E)** in the high- and low-risk groups through ESTIMATE. **(F)** Heatmap demonstrating differences in immune-related functions between the high- and low-risk groups via ssGSEA. (*, *P* < 0.05; **, *P* < 0.01; ***P < 0.001; ****P < 0.0001).

Afterwards, we carried out functional and pathway enrichment analysis of the differentially expressed genes between two groups, and picked the top ten givens in their respective ranges to be displayed with bubble plots (Supplementary Figures S3A–D). Upon scrutinizing the pathways and features of these enrichments we found that the prognostic model was most likely engaged in immune-related activities. As a result, we further made judgments about the m7G-related-lncRNA prognostic model’s immune-related functions and impacts. In terms of immune cell distribution, the current study had characterized the relative proportions of 22 immune cells in patients belonging to the high and low risk groups of ovarian cancer TCGA cohort by the use of the CIBERSORT algorithm (Figure 7A), which had a significantly higher proportion of infiltration of CD8 T cells, Tregs and M1 macrophages in the low-risk group, compared with the high-risk group. Simultaneously, the ESTIMATE algorithm assessed the immune score, stromal score, and estimate score between the two groups and revealed a statistically significant difference in the immune score, with the high-risk group having distinctly less immune scores than the low-risk group, which explicitly pointed to the presence of a worse immune microenvironment in the high-risk group with a dismal prognosis, which was in line with our aforementioned results (Figures 7B–D). Also, tumor purity of high risk group was above that of low risk group, while this did not statistically differ of, it still plausibly accounted for the poorer prognosis of the high risk group (Figure 7E). Subsequent analysis of the discrepancies in 13 immune functions among patients in the high- and low-risk groups with the ssGSEA method, the immune function heatmap results displayed remarkable differences with the high-risk group having significantly lower enrichment scores than the low-risk group for most of the immune functions, such as type I IFN response, cytolytic activity, parainflammation, check point and human leukocyte antigens (Figure 7F). These findings indicated that m7G-associated lncRNAs are intimately involved in tumor immunity and that they will be instrumental in guiding immunotherapy for OC patients.

Often referred to as TIDE to determine immunotherapy efficacy, previous investigations have confirmed that patients with higher TIDE scores have stronger likelihood of immune escape, inferior immunotherapeutic response, and unfavorable prognosis (Xie et al., 2023; Wei et al., 2022). And our outcomes displayed that the high-risk group having the comparatively higher TIDE scores exhibited weaker immunotherapy response rate, which was in line with the results of the analysis of immune cells and functions in the aforementioned section (Supplementary Figure S4A). Additionally, We investigated the expression levels of immune checkpoints in overall patients using m7G-related lncRNA model to verify the above mentioned differences in Check-point of immune function by ssGSEA method and found that there were indeed great differences between the two risk groups, which provided fresh ideas to guide us in the following personalized treatments (Supplementary Figure S4B). When it comes to therapy, the sensitivity of anticancer drugs was being compared between OC patients in high and low risk groups. The IC50 of cisplatin, paclitaxel, sorafenib, erlotinib, mitomycin C, midostaurin, lenalidomide, and nilotinib differed significantly with respect to each other between the high- and low-risk groups, suggesting that the m7G-related lncRNA risk model had the potential to predict chemotherapy sensitivity (Supplementary Figure S4C). Moving forward, those findings could contribute to the tailored therapy for OC patients.

### 3.8 Development ceRNA network and qRT-PCR verification m7G-related lncRNA prognostic

**FIGURE 8:**
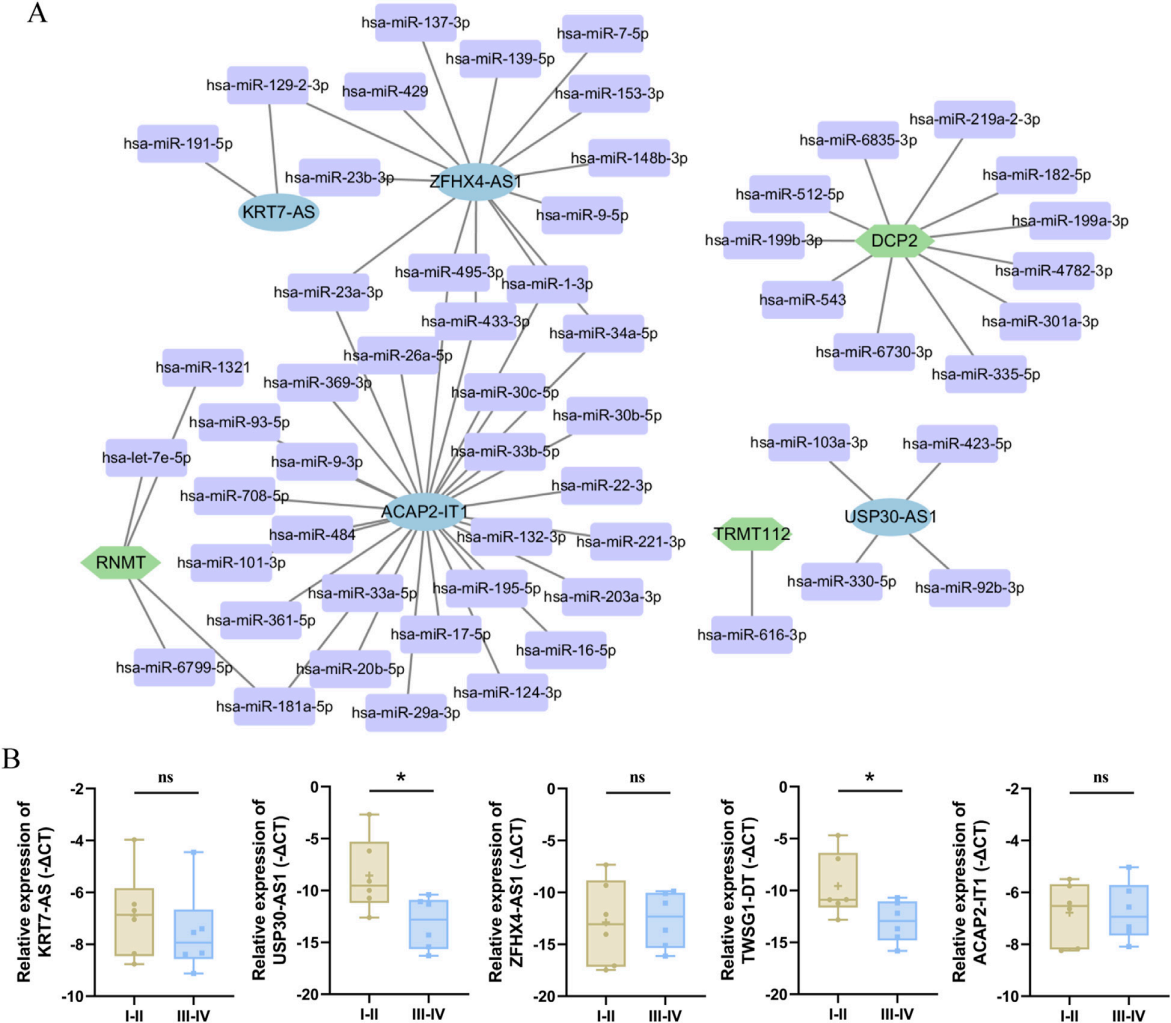
Construction a ceRNA network and verification the differential expression of 5 m7G-related lncRNAs. **(A)** The ceRNA network consisting of the m7G-related lncRNAs prognostic signature, the miRNAs with them, and aasociated m7G genes. Blue represents the m7G-related lncRNAs prognostic signature, purple represents miRNAs, green represents m7G genes. **(B)** The qRT-PCR experiment results of five m7G-related lncRNAs in early and late OC through the Mann–Whitney U test. The data are presented as mean ± SEM. (ns, not significant; *, *P* < 0.05).

Up to this point, we have sufficiently demonstrated the prognostic value and immune response of the five m7G-related lncRNAs. Then we attempted to construct a ceRNA regulatory network of m7G-related genes, m7G-related prognostic lncRNAs and miRNAs interacting with both of the above. The miRNAs interacting with the above lncRNAs and mRNAs were obtained through the Lncbase and miRWalk databases, respectively, as well as the intersections of the acquired miRNAs, excluding the scattered points without interactivity, and a ceRNA network including 4 m7G-associated lncRNAs, 3 m7G-regulated factors and 57 miRNAs was constructed and visualized using the cytoscape software (Figure 8A).

To further validate the differential expression levels of these five m7G-related lncRNAs, this was done in the early-stage and advance-stage lesions of ovarian cancer using RT-PCR assay, which was undertaken in response to the fact that advanced ovarian cancer denotes a higher stage of ovarian cancer with a poorer prognosis. Later on, this was followed by additional statistical analyses showing that only USP30-AS1 and TWSG1-DT displayed significant differences between the OC early and late stage tissues, while the other three lncRNAs(KRT7-AS, ZFHX4-AS1, and ACAP2-IT1) failed to have statistically significance shown, which could be due to the relatively minor specimen size of the present study (Figure 8B).

## 4 Discussion

As the most common malignant tumor in gynecology, the lethality of ovarian cancer is at the top of the three major female reproductive system cancers, and while surgery and chemotherapy with paclitaxel and platinum have made certain advances in the treatment of ovarian cancer, the overall prognosis of OC is still dismal, with approximately 70% of patients recurring and ultimately dying from the disease within 3 years (Li et al., 2023; Casey et al., 2024; Martincuks et al., 2024; Li et al., 2024; Ledermann et al., 2013). PARP inhibitors, which were once deemed therapeutically promising, are only beneficial for about 25% of patients with BRCA mutations, and anti-angiogenesis therapies do not lead to a meaningful improvement in the prognosis of ovarian cancer (Lheureux et al., 2023; Bao and Li, 2024). These limitations of clinical management have to urge us to identify more precise and effective therapeutic options to achieve more survival time and establish the better quality of life for ovarian cancer patients.

There are increasing literatures on the role of m7G as a novel RNA modification that should not be underestimated in malignant tumors, as well as lncRNAs have attracted wide attention in recent years for their ability to function through different identities. For our study, we sought to construct a risk model that could guide the prognosis of ovarian cancer patients, which consisted of five m7G-related lncRNAs (KRT7-AS, USP30-AS1, ZFHX4-AS1, ACAP2-IT1, and TWSG1-DT) could not only serve as an independent prognostic factor for ovarian cancer patients, but also it was indicative of instructing and predicting the overall survival of ovarian cancer. Combined with other clinicopathologic features of the clinical information, this risk signature still had unique advantages that were far superior to the conventional baseline profiles. In addition, we assessed the operational efficacy of this model by ROC analysis, and the AUC values were above 0.6 for all specific conditions, which further confirmed the exceptional prognostic precision of the five m7G-related lncRNAs.

On top of that, we also characterized the tumor mutation burden in the high- and low-risk groups and detected that there was definitely difference in TMB between the two risk groups. Following this, we conducted KM survival analysis for the high and low TMB groups, and patients in the high TMB group had a better prognostic survival. Combined with the risk score, we found that the survival prognosis of patients in the low-risk and high-TMB groups were the best among the four groups, which indicated that TMB could further help to predict the prognostic level of patients in the low-risk group, and also confirmed that the TMB analysis was meaningful in assisting in the assessment of prognosis of ovarian cancer patients. It may be that tumor mutation rates are more frequent in the low-risk group, which rendered treatments targeting the mutated genes more effective, leading to a higher survival rate for patients in the low-risk group.

Furthermore, we have analyzed the differentially expressed genes in the high- and low-risk groups and demonstrated the functional and pathway differences between the two groups. Comprehensively analyzing the enriched pathways and biological functions in KEGG and GO analysis, we noticed that most of the discrepant functions were mainly reflected in the immune aspects, such as allograft rejection, lymphocyte-mediated immunity, leukocyte-mediated immunity, adaptive immune response, and immune response-regulating signaling, which aroused our new thinkings, and at the same time, provided us with a brand-new perspective, whether we can take the advantage of this prognostic model to assess the immune landscape in OC patients, to formulate more accurate therapeutic protocols and to boost the immune responsiveness of ovarian cancer, so as to realize even more efficacious and personalized immune treatment, and consequently to achieve the clinical purpose of strengthening the prognosis as well as improving the survival status of the patients.

Accordingly, we specifically scrutinized the immune cell infiltration in the high- and low-risk groups with the help of two available algorithm and detected that the expression abundance of CD8 T cells and M1-type macrophages was obviously higher in the low-risk group than that in the high-risk group. CD8 T cells, as the central theme in immunotherapy, have been reported in a large number of literatures, and it was consistently demonstrated that there is a certain positive correlation between the number of CD8 T cells and the response to immunotherapy, as well as a sharp linear trend with the survival rate of malignant tumors (Fridman et al., 2012; van Elsas et al., 2024). M1-type macrophages are capable of carrying out a variety of anti-tumor functions such as phagocytosis and cytokine-mediated cell death in the tumor immune microenvironment, and their presence predictably reflects a favourable reaction to immune checkpoint inhibitor therapy (van Elsas et al., 2024; Mantovani et al., 2022). CD8 T cells derived resolvable elements can activate the aforementioned tumor killing function of M1-type macrophages, simultaneously these M1-type macrophages in turn expressed CXCL9 and CXCL10, which in positive feedback to the T cell infiltration, augmented the response to T cell immunotherapy, and consequently raised the overall survival of the patients (Tokunaga et al., 2018). These studies further validate the effectiveness of our model for steering immunotherapy, while we can subsequently dig deeper into some unreported new therapeutic concepts, which are expected to be full of innovative advances.

In addition to focusing on the differences in immune cells between the two groups, we also investigated immune function in the high and low risk groups. Several previous studies have confirmed that tolerance of type I IFN generation by tumor cells is the underlying mechanism of immune evasion and resistance to immunotherapy, and this theory further authenticates the accuracy of our experimental results, in the immune function analysis experiment, the response to type I IFN was attenuated in the high-risk group, which may be due to the above mentioned effects of immune evasion and immunotherapeutic resistance, which resulted in poor prognosis of ovarian cancer in the high-risk group (Miranda et al., 2024; Lawson et al., 2020). Tumor immunotherapy, including immune checkpoint inhibitors, can be effective in many malignant diseases, whereas its benefits in ovarian cancer are minimal, even though a high level of genomic instability and tumor mutation load exists in ovarian cancer patients, which is attributed to the reason why our study concluded that the immune response to the same immune checkpoint inhibitor varied in different OC patients (Kment et al., 2024; Wang ZB. et al., 2024). In the immunity heatmap, we can have observed that the scores of immune checkpoints in the high-risk group are distinctly lower than those in the low-risk group, which means that when confronted with the identical immune checkpoint treatment, patients in the high-risk group are not as well managed as those in the low-risk group. From another point of view, we will be able to customize the treatment according to the immune checkpoints which are specific to each of the high- and low-risk groups, and it is based on our constructed prognostic model that the expression levels of immune checkpoints CD160, CD276, and TNFRSF25 in the high-risk group are clearly higher than those in the low-risk group, which implied that inhibitors targeting the above mentioned immune checkpoints would be of better therapeutic utility in the high-risk group, and that the results would be of guidance for the development of the individualized immune therapy programs for ovarian cancer.

More than just research on immunotherapy, our model can also give some constructive opinions on the sensitivity to some anti-cancer drugs. Paclitaxel and platinum-based chemotherapeutic agents are the first choice of chemotherapy for ovarian cancer, according to this prognostic model, the IC50 of paclitaxel and platinum-based medicines is lower in the low-risk group, which points out that the low-risk group is more susceptible to the above mentioned drugs than the high-risk group, and it may be one of the reasons behind the better prognosis of the patients in the low-risk group.

Notwithstanding the important functions of our m7G-related lncRNA signature in assessing prognosis and instructing treatment, this study still has some limitations. First and foremost, while the study set up both experiment and validation groups, it was accomplished based on the information of the samples in the database, and was not verified in clinical samples with large samples. Moreover, we will continue to complete the evaluation of *in vitro* and *in vivo* experiments and molecular mechanism experiments if necessary, but the prognostic study of clinical samples has a long period of time, and for the time being, we are still in the process of collecting prognostic specimens, which does not have the required status for the study. Finally, albeit our study has discovered the function of m7G-related lncRNA, how lncRNA acts with m7G regulators and what is the mechanism of effect need to be further refined in the follow-up.

## 5 Conclusion

To summarize, we have constructed an m7G-associated lncRNA prognostic model involving five lncRNAs (KRT7-AS, USP30-AS1, ZFHX4-AS1, ACAP2-IT1, and TWSG1-DT), where the guiding value of this model for OS in ovarian cancer patients was measured and confirmed, as well as it can be functioned as an independent prognostic factor for OC patients. Meanwhile, we made the enrollment of the differential function of the model, focusing on the aspects of immune cells and immune function, and concluded that the risk signature could well evaluate the immune efficacy and immune response of OC patients. In addition, we have screened more scientific and effective therapeutic options for OC patients in terms of gene mutations, immune checkpoints, and small molecule drugs, which lays a robust foundation for the realization of precision management of ovarian cancer patients.

## Data Availability

The original contributions presented in the study are included in the article/Supplementary Material. Further inquiries can be directed to the corresponding author.

## Supplementary material

SUPPLEMENTARY TABLE S1 A list of 40 m7G regulators.

SUPPLEMENTARY FIGURE S1 The correlation between m7G regulators and differential expression lncRNA.

SUPPLEMENTARY FIGURE S2 Somatic mutation and TMB analysis. **(A, B)** The waterfall chart displaying the top 20 genes with high mutation frequency in the high-**(A)** and low-risk **(B)** groups based on the lncRNA signature. **(C)** TMB differences between high- and low-risk groups. **D,** KM curve analysis of OS in high and low TMB score groups. L-TMB means Low TMB and H-TMB refers to high TMB. **E,** KM survival analysis of OS stratified by tumor mutation burden and the m7G-related lncRNA model.

SUPPLEMENTARY FIGURE S3 Enrichment analysis based on differential expressed genes from the high- and low-risk subgroups. **(A)** Top ten of pathway enrichment in the KEGG database. **(B, D)** Top ten of GO function enrichment in terms of MF **(B)**, CC **(C)**, and BP **(D)**.

SUPPLEMENTARY FIGURE S4 The immunotherapy and candidate drugs prediction of risk subgroups. **(A)** The TIDE scores in different risk groups. The scores were compared by the Wilcoxon test. **(B)** The differentially expressed immune checkpoint gene in risk subgroups. The expression levels were compared by the Wilcoxon test. (**C)** The estimated IC50 of 8 representative anticancer drugs for high- and low-risk groups in OC patients. (ns, not significant; *, *P* < 0.05; **, *P* < 0.01; ****P* < 0.001; *****P* < 0.0001).
