# Supplementary figures and images for: Identification and validation of an m7G-related lncRNAs signature for predicting prognosis, immune response and therapy landscapes in ovarian cancer
Source: Front Genet. 2024 Oct 8;15:1466422. doi: 10.3389/fgene.2024.1466422 (PMC11493627; doi:10.3389/fgene.2024.1466422)

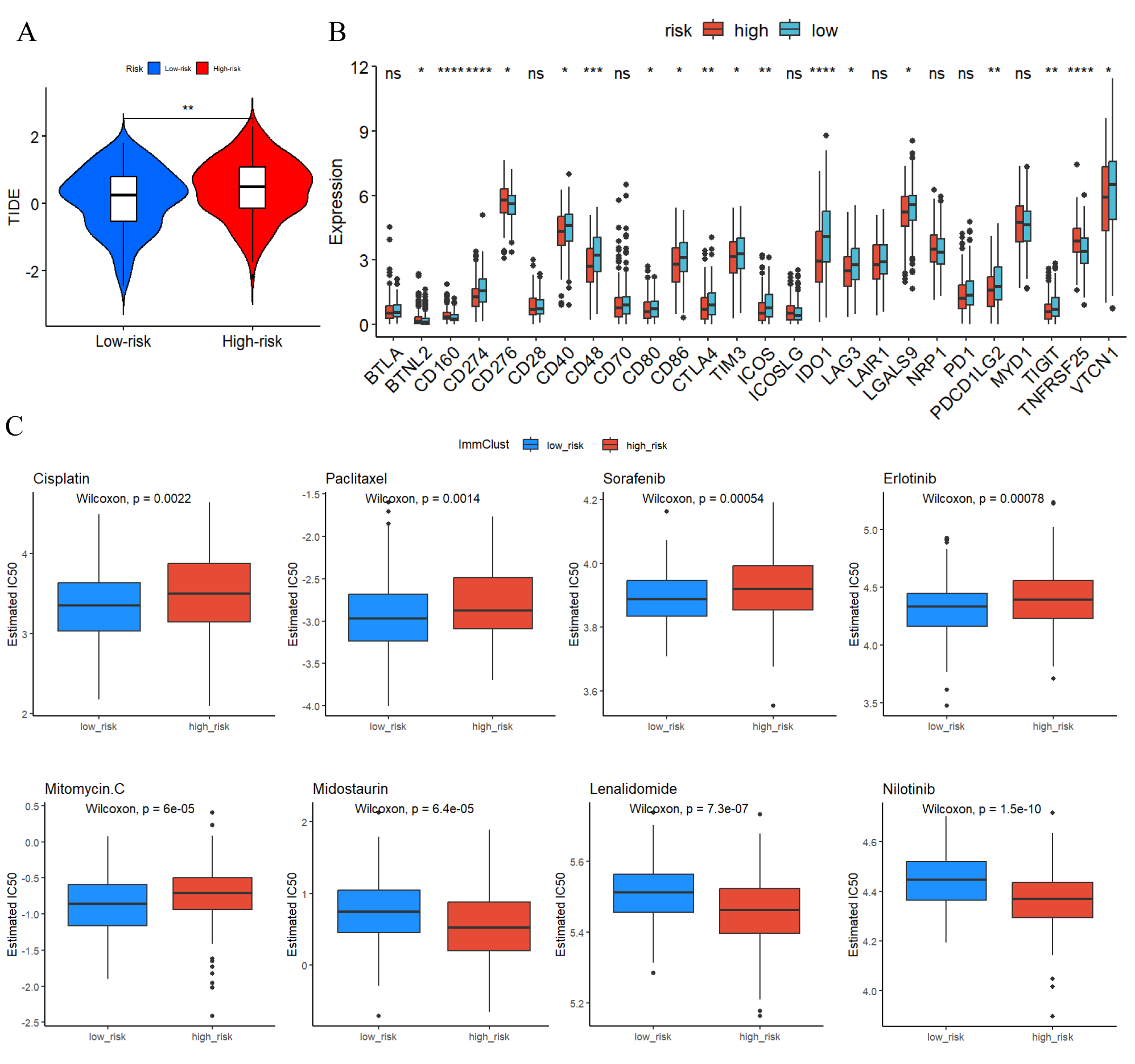

Supplement: Supplementary file 1 [file Image3.TIF]

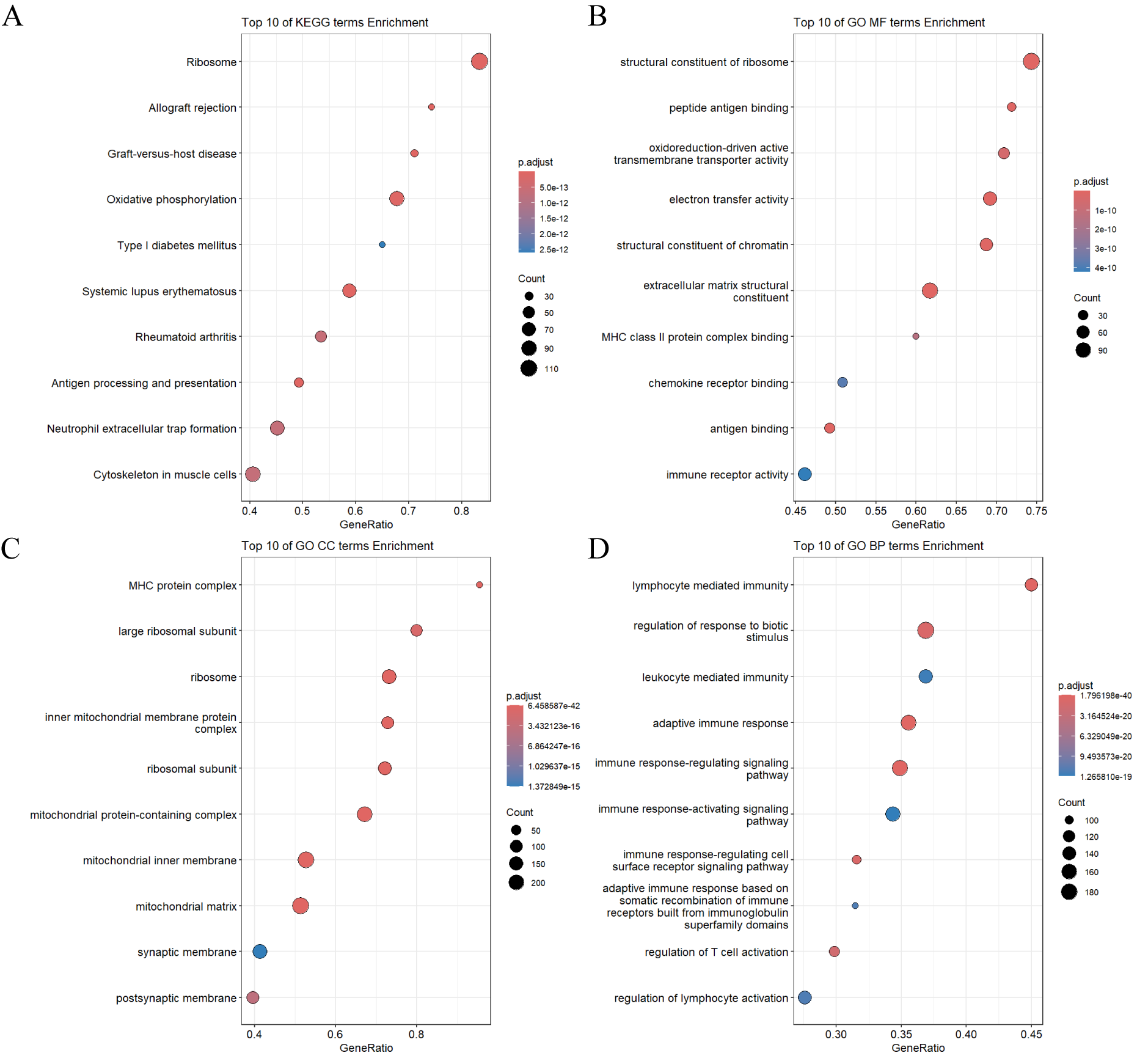

Supplement: Supplementary file 2 [file Image2.TIF]

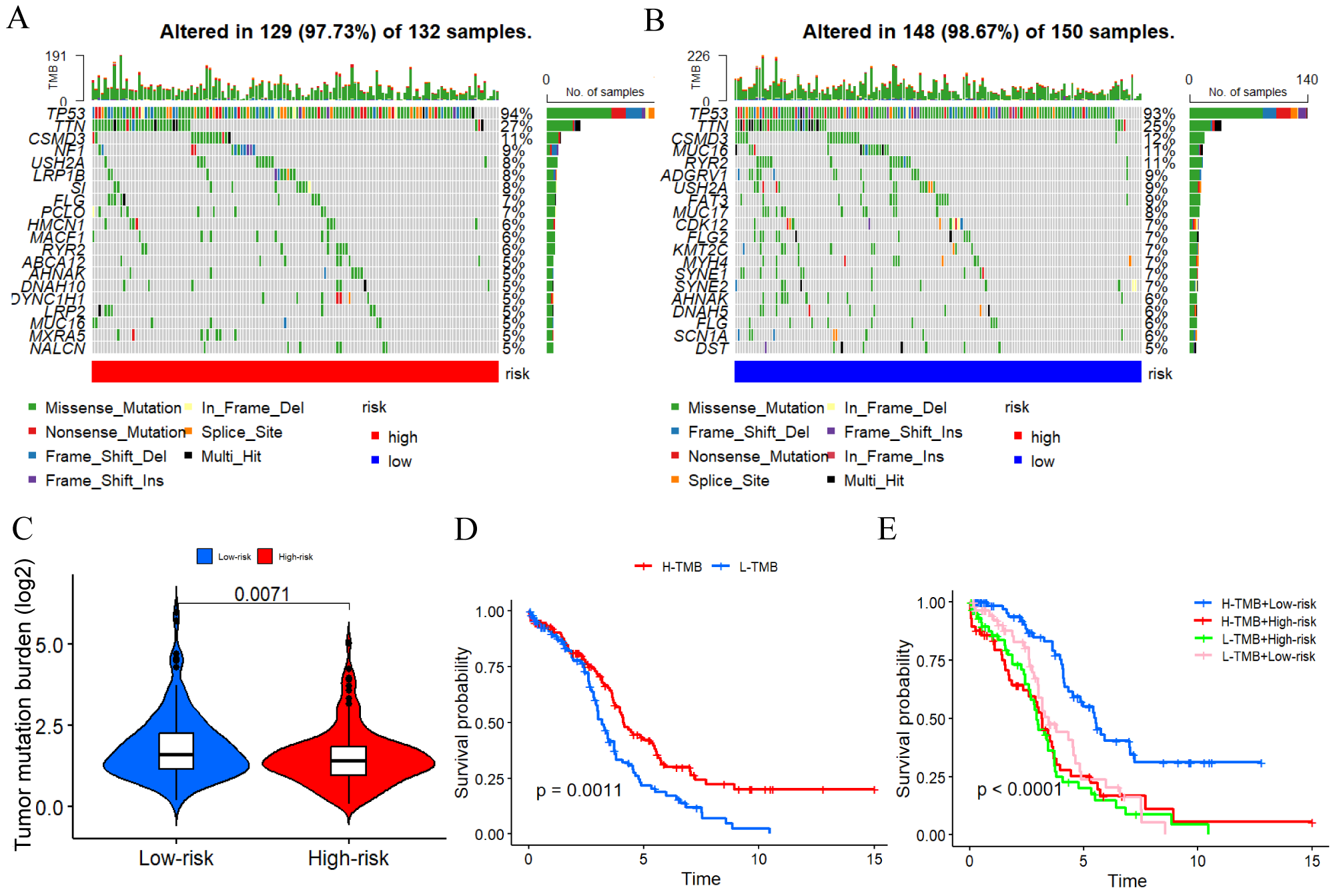

Supplement: Supplementary file 3 [file Image1.TIF]

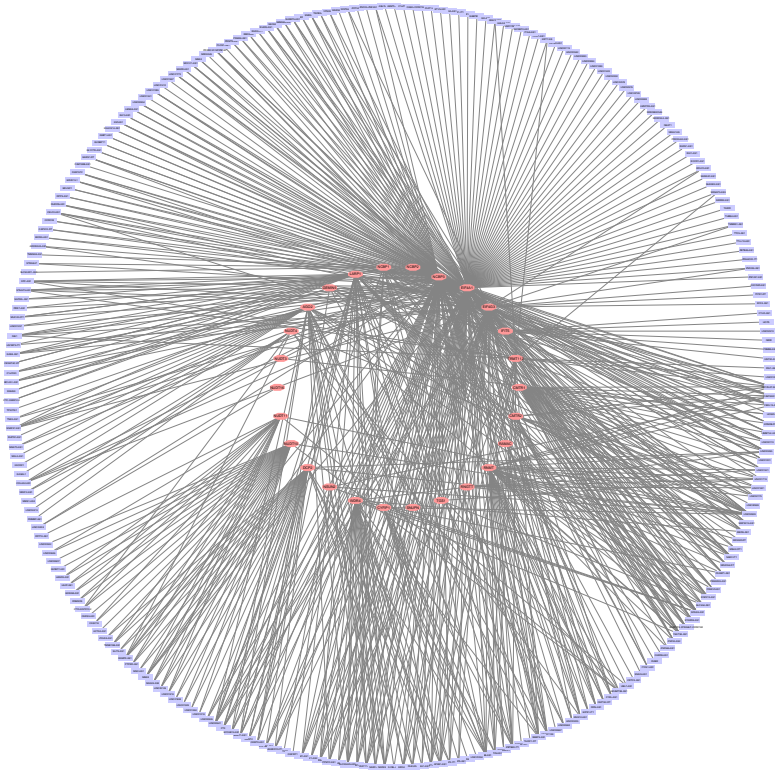

Supplement: Supplementary file 5 [file DataSheet1.PDF]
