# Supplementary material for: Identification and validation of an m7G-related lncRNAs signature for predicting prognosis, immune response and therapy landscapes in ovarian cancer
Source: Front Genet. 2024 Oct 8;15:1466422. doi: 10.3389/fgene.2024.1466422 (PMC11493627; doi:10.3389/fgene.2024.1466422)
Supplement: Supplementary file 4 [file Table2.DOCX]

Supplement Table S2 The primer sequences of m7G-related lncRNA and Actin

| Gene name | Sequence of primer (5’- 3’) |
| --- | --- |
| KRT7-AS Forward | CTCTTTCTGCTGGAGGAATACAA |
| Reverse | GCCGTTACCCACTCACTAATAC |
| USP30-AS1 Forward | AGCAATAGCTGACGGACCAC |
| Reverse | TGAAAACCAAGCAGCCCCA |
| ZFHX4-AS1 Forward | CTGCCTCTTCCCGCTTTAAT |
| Reverse | GCCAGCCGGTGATTTGATA |
| ACAP2-IT1 Forward | GTAGCAAGCACAAGTTGGGAATCG |
| Reverse | GGACGGCAGCACATGAGACC |
| TWSG1-DT Forward | TCGTTTGCCGTCAGAGGTGT |
| Reverse | ACATGTGTGCACTTACCAAGCA |
| Actin Forward | CCTGGCACCCAGCACAAT |
| Reverse | GGGCCGGACTCGTCATAC |
